# Ichthyol in Erysipelas

**Published:** 1895-08

**Authors:** 


ICHTHYOL IN ERYSIPELAS.—Dr. J. Abbott Cantrell (*Phil. Polyclinic*, 1895, IV., p. 245).—In his hospital and private practices, the author has had the opportunity of treating in all about one hundred cases of erysipelas with ichthyol. He used it in a number of different ways. In about one-fourth of the cases he made use of the drug in 33 per cent. aqueous solution, and found that if the case was a mild one, this was entirely sufficient to produce a cure in a short time; but when the disease had existed for some time, he prescribed the drug in another manner.

In about half of the cases he applied the ichthyol in 50 per cent. ointment (with petrolatum). This quickly brought about a cure; but to obviate the disagreeable nature of the preparation, the author tried to employ this medicament in other forms. In about one-quarter of the cases he used a mixture of equal parts of ichthyol, ether, and collodion. This acted equally well; but the collodion dried on the part, and it was necessary to remove it before each new application so as to allow the latter to reach the diseased area.

In the remaining fourth of the cases treated, Dr. C. prescribed equal parts of ichthyol and glycerine, with the result that the patients suffered no discomfort from the treatment, but were much comforted, while the disease was held in check and cured in as short a time as by the above methods. Of all the methods of application of ichthyol in erysipelas, the author therefore, prefers that with glycerine.

